# Supplementary material for: Effects of Free-Range Systems on Muscle Fiber Characteristics and Welfare Indicators in Geese
Source: Animals (Basel). 2025 Jan 22;15(3):304. doi: 10.3390/ani15030304 (PMC11816273; doi:10.3390/ani15030304)
Supplement: Supplementary file 1 [file animals-15-00304-s001.zip › animals-3352710-supplementary.pdf]

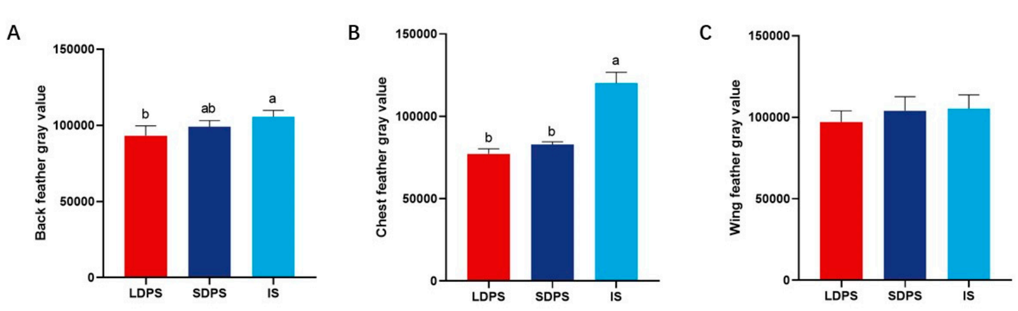

Figure S1: ImageJ was used to analyze the grayscale of Yangzhou goose feather images. A: Back feather gray value, B: Chest feather gray value, C: Wing feather gray value. LDPS, long-distance pasture system; SDPS, short-distance pasture system; IS, indoors system. Statistically significant differences are indicated by different letters.
